# Sequence and structural insights of monoleucine-based sorting motifs contained within the cytoplasmic domains of basolateral proteins

**DOI:** 10.3389/fcell.2024.1379224

**Published:** 2024-03-01

**Authors:** Sarah J. Harmych, Claiborne W. Tydings, Jens Meiler, Bhuminder Singh

**Affiliations:** ^1^ Department of Medicine, Vanderbilt University Medical Center, Nashville, TN, United States; ^2^ Department of Cell and Developmental Biology, Vanderbilt University, Nashville, TN, United States; ^3^ Department of Chemistry and Center for Structural Biology, Vanderbilt University, Nashville, TN, United States; ^4^ Institute for Drug Discovery, Leipzig University Medical School, Leipzig, Germany; ^5^ Epithelial Biology Center, Vanderbilt University Medical Center, Nashville, TN, United States

**Keywords:** epithelial polarity, polarized protein trafficking, basolateral sorting motif, clathrin adaptor proteins, monoleucine-based motif

## Abstract

Delivery to the correct membrane domain in polarized epithelial cells is a critical regulatory mechanism for transmembrane proteins. The trafficking of these proteins is directed by short amino acid sequences known as sorting motifs. In six basolaterally-localized proteins lacking the canonical tyrosine- and dileucine-based basolateral sorting motifs, a monoleucine-based sorting motif has been identified. This review will discuss these proteins with an identified monoleucine-based sorting motif, their conserved structural features, as well as the future directions of study for this non-canonical basolateral sorting motif.

## 1 Introduction

**FIGURE 1:**
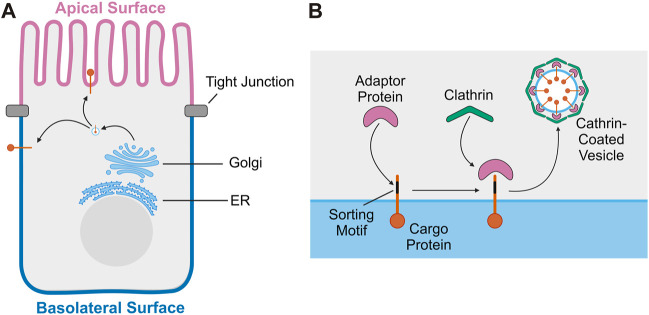
Trafficking of transmembrane proteins in polarized epithelial cells. **(A)** Overview of transport of transmembrane proteins from the Golgi to the plasma membrane in polarized epithelial cells. **(B)** Diagram of the sorting and loading of transmembrane proteins into vesicles at the Golgi. Sorting motif of cargo protein is recognized by adaptor protein. The adaptor protein then recruits clathrin, facilitating the formation of a clathrin-coated vesicle which will then transport the cargo protein to the plasma membrane.

Polarized epithelial cells line body cavities and organs throughout the body, forming a barrier critical for organ function. The structure and function of polarized epithelial cells depends on maintaining biochemically distinct apical and basolateral membrane domains with asymmetric distributions of proteins and lipids (Apodaca et al., 2012; Mostov et al., 2003). Membrane lipid composition has been shown to play a key role in both the establishment and maintenance of epithelial polarity and protein trafficking (Bugda Gwilt and Thiagarajah, 2022). Epithelial cells achieve asymmetric protein distribution primarily through the selective targeting of newly synthesized proteins to either the apical or basolateral membrane (Matter, 2000). Newly synthesized transmembrane proteins are inserted into the endoplasmic reticulum and transit through the Golgi apparatus, eventually reaching the trans-Golgi network (TGN) (Mostov et al., 2003). At the TGN, transmembrane proteins are incorporated into vesicles that deliver them either directly to the plasma membrane or to endosomes and then to the cell surface (Ang et al., 2004) (Figure 1A). Alternatively, asymmetric distribution of proteins in polarized epithelial cells is achieved through selective retention at the plasma membrane. In this mechanism, newly synthesized proteins are delivered both to the apical and basolateral membrane, but endocytosed from or recycled back to a specific cell surface, leading to a steady state polarized distribution (Matter, 2000).

Short peptide motifs or post-translational modifications serve as signals that facilitate the selective targeting of proteins to a specific membrane domain in polarized epithelial cells (Cao et al., 2012; Bonifacino, 2014). Basolateral sorting motifs are well defined and are usually a short amino acid sequence in the cytoplasmic domain of the protein (Cao et al., 2012). Like basolateral sorting motifs, apical sorting motifs are also identified within short amino acid sequences, or post-translational modifications, including a glycosylphosphatidylinositol (GPI) anchor or N- and O-linked glycosylation (Cao et al., 2012; Weisz and Folsch, 2020). Apical sorting motifs have been identified in the extracellular, transmembrane, or cytoplasmic region of apical proteins and lack a unifying mechanism to direct apical localization (Weisz and Folsch, 2020). Basolateral localization is often directed by adaptor proteins, which recognize sorting motifs within cargo proteins to concentrate them into a small region of the TGN membrane (Cao et al., 2012). Adaptor proteins then recruit clathrin to the membrane, facilitating the generation of a clathrin-coated vesicle containing the cargo proteins to be brought to the plasma membrane (McMahon and Boucrot, 2011; Park and Guo, 2014) (Figure 1B). Using this mechanism, apical and basolateral proteins are sorted into separate vesicles and transported to their appropriate destination. Disruption of this process can impact epithelial function and cause disease (Stein et al., 2002).

## 2 Basolateral sorting in polarized epithelial cells

Basolateral sorting motifs most often reside in the cytoplasmic domain of the protein and are usually recognized by members of the clathrin adaptor protein (AP) family (Fölsch, 2005). These heterotetrameric adaptor complexes are responsible for recognizing and binding to cargo molecules and recruiting clathrin to generate a clathrin-coated vesicle for transport (McMahon and Boucrot, 2011). Basolateral proteins in polarized epithelial cells are most often recognized by the epithelial cell-specific variant of AP-1, AP-1B, but there are known AP-1B independent pathways for basolateral targeting (Fölsch, 2005). AP complexes specifically mediate the trafficking of proteins within one compartment of the cell, such as AP-1B mediates trafficking from the TGN to the basolateral membrane. Selective recruitment of AP complexes to the membrane is directed by lipid composition of the membrane and GTPases present on the membrane (Park and Guo, 2014). In the case of AP-1, phosphatidylinositol 4 phosphate (PI(4)P) serves as the lipid directing its localization and ADP-ribosylation factor 1 (Arf1) serves as the GTPase (Ren et al., 2013; Park and Guo, 2014). Basolateral sorting motifs have been well characterized and often fall into two families, tyrosine-based and dileucine-based. Both motifs are recognized by AP complexes, specifically AP-1, which facilitates their incorporation into clathrin-coated vesicles destined for the basolateral surface (Robinson, 2004).

Tyrosine-based sorting motifs are characterized by the presence of a critical tyrosine residue and follow the general form YXXΦ (Y is tyrosine, X is any amino acid, and Φ is a bulky hydrophobic amino acid) (Bonifacino and Dell’angelica, 1999). While most well-known for targeting transmembrane proteins to the basolateral membrane, tyrosine-based sorting motifs also mediate rapid internalization of proteins from the cell surface and targeting proteins to other cellular compartments, such as lysosomes (Marks et al., 1997). The ability of tyrosine-based sorting motifs to differentially direct protein localization is mediated by their ability to interact with several members of the AP family, including AP-1B, which facilitates its role in directing proteins to the basolateral membrane (Bonifacino and Dell’angelica, 1999). Tyrosine-based sorting motifs were first determined to interact with the μ2 subunit of AP-2. The binding pocket for tryosine-based sorting motifs on the μ2 domain has a banana-shaped structure with binding pockets for both the Y and Φ residues of the sorting motif (Owen and Evans, 1998). The X residues or residues adjacent to the motif may provide other points of contact, but the Y and Φ residues are critical (Marks et al., 1997). Based on sequence alignment, the μ2 subunit residues important for this interaction are conserved across the μ subunits of the other AP family members, indicating that they interact with tyrosine-based sorting motifs in the same way. Interacting with different complexes in the AP family allows tyrosine-based sorting motifs to facilitate transport to multiple compartments within the cell (Bonifacino and Dell’angelica, 1999).

The other major group of basolateral sorting motifs is dileucine-based. These motifs are characterized by the presence of critical leucine-leucine or leucine-isoleucine residues and come in two forms defined by the consensus motifs DXXLL and [DE]XXXL[LI]. (D is aspartic acid, E is glutamic acid, X is any amino acid, L is lysine, and I is isoleucine) (Janvier et al., 2003; Bonifacino, 2014). DXXLL motifs mediate intracellular sorting between the TGN and endosomes while [DE]XXXL[LI] motifs target proteins to endosomes, lysosomes, and the basolateral membrane. The leucine-leucine or leucine-isoleucine pairs are critical elements for the motifs while the upstream aspartic acid or glutamic acid is often important but not essential for function (Janvier et al., 2003). Even though the AP subunit interacting with tyrosine-based sorting motifs is well defined, the identity of the AP subunit that recognizes dileucine motifs is controversial. Mutational analysis has demonstrated that the binding site for dileucine motifs is on the β subunit of AP-1 (Rapoport et al., 1998). More recently, yeast two-hybrid analysis was unable to detect a strong interaction between the dileucine motifs and any of the AP subunits, which suggests a requirement for more than one subunit. In agreement with this, yeast-three hybrid experiments showed recognition by a combination of the γ and σ1 subunits of AP-1 and the δ and σ3 subunits of AP-3. This study was unable to determine whether the interaction requires binding sites on both the subunits or that the interaction between the subunits and the motif is more complex (Janvier et al., 2003). Co-crystallization of eight different dileucine-based sorting motif peptides with AP-2 further supports the idea of more than one subunit being involved in the interaction with dileucine-based sorting motifs, finding the binding site to be on the α-σ2 heterodimer (Kelly et al., 2008).

Outside of the AP complexes, PDZ (PSD95, DlgA, ZO-1) proteins have also been shown to play a role in the polarized distribution of proteins in epithelial cells. PDZ proteins contain an 80–90 amino acid PDZ domain which bind to PDZ target motifs on the C-terminal end of target proteins (Muth and Caplan, 2003). The most common PDZ target motif follows the sequence X- S/T - X - Φ (X is any amino acid, Φ is a hydrophobic residue, typically I, L, V, or F). Unlike AP complexes, which bind internal sorting motifs, PDZ proteins typically bind C-terminal motifs, engaging with the carboxylate moiety during peptide binding (Amacher et al., 2020). While AP complexes are highly specific for certain sorting motifs, PDZ binding interactions are considered promiscuous. Due to the shallow nature of the PDZ binding cleft, there are a limited number of stereochemical restrictions on peptide recognition and binding, resulting in a number of PDZ domains being able to bind the same target (Amacher et al., 2020). PDZ domains have been shown to play a role in the selective retention of basolateral proteins in polarized epithelial cells, contributing to these proteins’ basolateral distribution. For example, the PDZ protein LIN-7 has been implicated in the selective retention of the GABA transporter BGT-1 (Perego et al., 1999), the proteoglycan syndecan-1 (Maday et al., 2008), and the receptor tyrosine kinase, ErbB2 (Shelly et al., 2003). PDZ proteins have also been implicated in the polarized distribution of apical proteins (e.g., CFTR; cystic fibrosis transmembrane conductance regulator), while AP complexes have only been implicated in the trafficking of basolateral proteins (Swiatecka-Urban et al., 2002).

Other less common sorting motifs have important implications for the localization of basolateral proteins. One of these motifs is a leucine-valine di-hydrophobic pair. This sorting motif was first identified in the cytoplasmic domain of CD44 and was shown to mediate its localization to the basolateral membrane in polarized epithelial cells (Sheikh and Isacke, 1996). A di-hydrophobic valine-leucine pair has also been shown to direct the basolateral localization of the MHC class I chain-related MICA molecule (Suemizu et al., 2001) and the basolateral localization of CD1d (Rodionov et al., 2000). CD1d is also an example of a basolateral protein whose cytoplasmic tail contains multiple sorting motifs. The cytoplasmic tail of CD1d contains the di-hydrophobic valine-leucine pair and a tyrosine-based sorting motif, both of which contribute to the basolateral localization of the protein (Rodionov et al., 2000). The cytoplasmic domain of epidermal growth factor receptor (EGFR) contains two functionally redundant basolateral sorting motifs, a dileucine-based sorting motif and a polyproline core-based sorting motif (He et al., 2002). EGFR ligands transforming growth factor α (TGFα) (Dempsey and Coffey, 1994) and betacellulin (Singh et al., 2015) also contain two basolateral sorting determinants. In the case of another EGFR ligand, epiregulin (EREG), when its basolateral motif is removed, EREG shifts to predominantly apical localization, suggesting the presence of a competing, recessive apical sorting motif in EREG (Singh et al., 2013).

While many basolateral proteins contain aforementioned sorting motifs, some lack standard identifying factors for basolateral sorting. Studies involving sequential cytoplasmic domain truncation mutants and site directed mutagenesis have revealed six proteins with a monoleucine-based basolateral sorting motif. These sorting motifs are characterized by a single leucine residue and an N-terminal acidic amino acid cluster with 3-5 residues between them. The remainder of this review will focus on the monoleucine-based sorting motif.

## 3 Proteins with identified monoleucine-based sorting motif

### 3.1 Stem cell factor (EDDXXXXXL)

A basolateral monoleucine-based sorting motif was first identified in stem cell factor (SCF) (Wehrle-Haller and Imhof, 2001). SCF is the ligand for the receptor tyrosine kinase c-Kit. Binding of SCF to c-Kit leads to the activation of signaling pathways involved in cell survival, migration, and proliferation. SCF belongs to a family of transmembrane growth factors with highly conserved cytoplasmic domains (Lennartsson and Rönnstrand, 2012). Studies of an SCF mutant with an altered cytoplasmic domain suggested localization and secretion from the basolateral membrane is critical for SCF function (Wehrle-Haller and Weston, 1999). The cytoplasmic domain of SCF lacks the standard tyrosine- or dileucine-based basolateral sorting motifs, but site-directed mutagenesis revealed a critical monoleucine residue and N-terminal cluster of acidic amino acids (Wehrle-Haller and Imhof, 2001). Substitution of the leucine residue with an alanine led to the apical localization of SCF in Madine-Darby Canine Kidney (MDCK) cells. Mutation of the acidic amino acid cluster N-terminal to the monoleucine led to a reduced efficiency of SCF basolateral targeting but did not recapitulate the complete apical localization caused by mutation of the leucine residue (Wehrle-Haller and Imhof, 2001).

### 3.2 CD147 (EDDXXXXXL)

Retinal pigment epithelial cells (RPE) display several plasma membrane proteins at the apical surface that typically localize to the basolateral surface in other epithelial cells, one of which is CD147 (Deora et al., 2004). In RPEs, CD147 shifts from a primarily basolateral localization in newborn rats, to a primarily apical distribution in adult rats. This shift suggests that the basolateral sorting motif in CD147 is suppressed or no longer recognized by RPE cells in mature rats (Marmorstein et al., 1998). The cytoplasmic domain of CD147, like SCF, lacked the standard tyrosine- or dileucine-based basolateral sorting motifs, which led to site-directed mutagenesis of the cytoplasmic domain to identify the sorting motif. Analysis of CD147 mutants revealed a monoleucine residue critical for basolateral targeting (Deora et al., 2004). When mutated, loss of this monoleucine led to almost complete apical localization of CD147. Unlike the sorting motif previously identified in SCF, basolateral targeting of CD147 did not require an acidic N-terminal amino acid cluster (Deora et al., 2004). Removal of a cluster of acidic amino acids N-terminal to the monoleucine residue resulted in apical mistrafficking, but replacement of these residues with alanine residues rescued normal localization. This suggests that these residues are not directly interacting with sorting machinery but instead are necessary for proper spacing within the cytoplasmic domain (Deora et al., 2004). Since the monoleucine-based sorting motif has only recently been identified, an adaptor protein recognizing this motif and facilitating its incorporation into clathrin-coated vesicles has not been determined. Basolateral localization of CD147 is not altered in cells which do not express AP-1B, the canonical basolateral adaptor protein, indicating that AP-1B may not mediate the trafficking of CD147 in polarized epithelial cells (Deora et al., 2004).

### 3.3 Amphiregulin (EEXXXL)

Amphiregulin (AREG) is a member of the epidermal growth factor (EGF)-like ligand family (Singh and Coffey, 2014). These ligands are produced as single-pass transmembrane proteins that, following delivery to the plasma membrane, are cleaved to produce a soluble signaling molecule. These soluble ligands then bind and activate members of the ErbB receptor family, including EGFR (Singh et al., 2016). These receptors activate signaling pathways that regulate processes such as cell growth, proliferation, and migration and are critical for epithelial tissue homeostasis (Wieduwilt and Moasser, 2008). Studies of both EGFR and some of the EGF-like ligands has demonstrated the importance of basolateral localization for their proper signaling (Kuwada et al., 1998; Singh and Coffey, 2014). AREG was shown to localize to the basolateral membrane in polarized epithelial cells in a cytoplasmic domain-dependent manner (Brown et al., 2001). Further investigation of the cytoplasmic domain revealed a monoleucine-based basolateral sorting motif which when removed lead to a nonpolarized localization pattern (Gephart et al., 2011). While mutation of both the monoleucine and N-terminal cluster of amino acids led to altered localization, substitution of the three intervening amino acids did not alter localization. These intervening amino acids present a key difference between the previously identified monoleucine-based sorting motifs of SCF and CD147, which both had five amino acids between the monoleucine and acidic amino acids while the sorting motif of AREG has only three residues (Gephart et al., 2011). Knockdown of AP-1B in polarized epithelial cells led to an altered steady state localization of AREG but did not impact polarized delivery of newly synthesized molecules (Gephart et al., 2011). This suggests a role for AP-1B in the recycling of AREG to the basolateral surface but not in the trafficking of AREG from the TGN to the plasma membrane, although it has not been determined if recycling by AP-1B is also mediated by the monoleucine-based sorting motif or by another yet unidentified motif.

### 3.4 Betacellulin (EEXXXL)

Betacellulin (BTC) is also an EGF-like ligand that binds and activates EGFR and ErbB4 (Singh et al., 2016). Like previously studied EGF-like ligands, the steady state localization of BTC is also basolateral. Removal of the cytoplasmic domain led to a nonpolarized steady state distribution of BTC and the formation of lateral lumens (Singh et al., 2015). The cytoplasmic domain of BTC contained a monoleucine-based sorting motif like AREG, with a critical monoleucine residue, N-terminal cluster of acidic amino acids, with three amino acids in between. Analysis of sequential cytoplasmic domain truncation mutants of BTC showed that loss of a cysteine-rich motif at the beginning of the cytoplasmic domain further disrupted the basolateral-sorting specificity of BTC (Singh et al., 2015). While substitution of this cysteine-rich motif alone did not alter the localization of full length BTC, combining this mutation with mutation of the monoleucine-based sorting motif led to further loss of basolateral BTC compared to full length BTC with only the monoleucine-based motif mutated. This suggests that both motifs play a role in the basolateral localization of BTC, which was not seen in the other three proteins with a monoleucine-based sorting motif. Like other EGF-like ligands, loss of polarized distribution of BTC had significant impacts on cell growth and development (Singh et al., 2015). Cells expressing an apical mutant of BTC developed lateral lumens, indicating that mistrafficked BTC impacted the maturation of polarized epithelial cell layers. A mutation causing mistrafficking of BTC has been observed in human cancers and confers a growth advantage to the cells, suggesting mistrafficking of EGF-like ligands can have clinical implications (Singh et al., 2015). Mistrafficking of another EGFR ligand, epiregulin, led to epithelial transformation *in vivo* and loss of polarity *in vitro* (Singh et al., 2013; Singh et al., 2021).

Sequence analysis of heparin-binding EGF-like growth factor (HB-EGF), another EGF-like ligand, has revealed the presence of a putative monoleucine-based sorting motif. Given the important regulatory role trafficking plays for other EGF-like ligands, the localization of HB-EGF and the potential role of this motif in directing its trafficking warrant further study.

### 3.5 Na^+^/I^−^ symporter (EEXXXL)

The sodium/iodide symporter (NIS) is a protein expressed at the basolateral membrane of thyroid follicular cells and mediates the concentration of iodide for the generation of thyroid hormones. Uptake of radioiodine is used to treat thyroid cancer and relies on both the proper function and localization of NIS (Ravera et al., 2017). Thyroid tumors often become resistant to this treatment method through loss of proper targeting of NIS to the plasma membrane rather than loss of NIS function, making the mechanism of NIS trafficking a potential target for cancer therapy (Martín et al., 2019). Removal of the cytoplasmic domain of NIS led to a significant reduction in the amount of NIS at the plasma membrane compared to wild-type NIS. A monoleucine-based sorting motif was identified in the cytoplasmic domain that, when altered, led to NIS being localized primarily at the apical membrane. This altered localization reduced the accumulation of iodide in the cells (Martín et al., 2019). The trafficking of NIS to the basolateral membrane follows a clathrin-dependent mechanism in which both the AP-1A and AP-1B adaptor complexes can mediate its sorting in the TGN and recycling endosomes (Martín et al., 2019; Koumarianou et al., 2022).

### 3.6 Matriptase (EEXXXXL)

Matriptase is an epithelial serine-protease whose expression has been seen in a variety of human tissues. Translated as a zymogen, matriptase requires a complicated series of steps to become active, including proteolytic cleavage and interaction with its cognate inhibitor (List et al., 2006). The basolateral localization of matriptase is important for both its proper activation and function, where these events take place (Tseng et al., 2020). Like most basolateral proteins, removal of the cytoplasmic domain of matriptase leads to a nonpolarized localization in MDCK cells (Murai et al., 2009). When a matriptase mutant lacking the cytoplasmic domain was expressed in keratinocytes, it accumulated in the nucleus and perinuclear area rather than at the plasma membrane like WT matriptase (Tseng et al., 2020). While complete removal of the cytoplasmic domain led to decreased plasma membrane delivery, a mutant with an altered monoleucine-based sorting motif was still delivered to the plasma membrane but showed a nonpolarized distribution. This suggests that while the entire cytoplasmic domain of matriptase plays a role in its delivery to the plasma membrane, the monoleucine-based sorting motif alone dictates preferential localization to the basolateral membrane (Tseng et al., 2020). Time-lapse video microscopy demonstrated that alterations to this motif impaired the movement of matriptase into the correct transport vesicles. The identification of the monoleucine-based sorting motif of matriptase introduced another variation of this motif. Previously identified motifs contained either three or five residues between the monoleucine and the cluster of acidic amino acids while the matriptase motif has four (Tseng et al., 2020).

## 4 Discussion

Disruption of normal protein localization has significant impacts on function, making their trafficking a critical regulatory mechanism to study. While the monoleucine-based sorting motif is a more recent discovery than other basolateral sorting motifs, it warrants the same detailed study as tyrosine- and dileucine-based sorting motifs. Altered trafficking of proteins directed by a monoleucine-based sorting motif, such as NIS and BTC, has been shown to play a role in disease (Singh et al., 2015; Martín et al., 2019).

Recent studies of dominant nephrogenic diabetes insipidus (NDI) have demonstrated a role in the development of disease for a monoleucine-based sorting motif (Kamsteeg et al., 2003). In NDI, water reabsorption in the kidneys is diminished due to mutations of the aquaporin-2 (AQP2) water channel. In the case of recessive NDI, AQP2 mutants are misfolded and retained in the ER (Gao et al., 2020). But in dominant NDI, a functional AQP2 channel is expressed on the plasma membrane and is localized to the basolateral membrane instead of the apical membrane. A frameshift mutation in the AQP2 gene alters the cytoplasmic domain and introduces a leucine residue that when mutated restores the apical localization of AQP2. Unlike the other monoleucine-based sorting motifs identified, this motif lacks glutamic acids upstream of the leucine (Kamsteeg et al., 2003). This suggests, that like dileucine based sorting motifs, the critical residue is the leucine, and the glutamic acid residues are not essential to proper localization (Janvier et al., 2003). The identification of this motif in mutant AQP2 demonstrates that the monoleucine-based sorting motif is dominant since it overrides any apical sorting motif present in WT AQP2. It also provides a different role for the motif: instead of being altered in disease, introducing the motif can lead to the development of disease.

There is also potential role for monoleucine-based sorting motifs in the trafficking of neuronal proteins. Glycoprotein M6a (Gpm6a) is expressed in the neurons of the central nervous system, participating in filopodia formation, neurite expression, and synaptogenesis (Alfonso et al., 2005). Analysis post-removal of the N- and C- termini individually indicated that the C-terminus is essential for the role of Gpm6a in filopodia formation (Rosas et al., 2018). One explanation for this change in filopodia formation is that the C-terminus is needed to interact with cytosolic proteins needed for this process to occur. The other is that loss of the C-terminus impacts trafficking of Gpm6a in neurons. Alanine-scanning mutagenesis revealed three residues, K250, E258, and E259, that were necessary for filipodium formation (Rosas et al., 2018). E258/E259 are potentially apart of the monoleucine-based sorting motif EEQEL in the cytoplasmic C-terminus. Colocalization studies showed that deletion of the C-terminus diminished the association of Gpm6a with clathrin. This further supports the possibility that the C-terminus, and the monoleucine-based sorting motif within it, are involved in the trafficking of Gpm6a in neurons, and that alterations to this trafficking impact the function of Gpm6a (Rosas et al., 2018). More research needs to be done to confirm the role of the potential monoleucine-based sorting motif in Gpm6a trafficking, but this study suggests future work on monoleucine-based sorting motifs should look outside of polarized epithelial cells for other proteins relying on this motif to properly function.

Further study of monoleucine-based sorting motifs will likely involve the identification of more proteins containing this motif. This will require sequence analysis to identify putative monoleucine-based sorting motifs followed by analysis of the impact of mutations on the localization of these proteins to confirm the motif’s role in trafficking. One major remaining question is what the role of the amino acids lying between the monoleucine and acidic amino acid cluster is. The number of residues occupying this space ranges from three to five. Mutation of these residues in AREG does not impact localization (Gephart et al., 2011), suggesting they are not directly involved in the interaction between this motif and an adaptor protein. Future studies of the monoleucine-based sorting motif also need to include both *in vivo* and 3D studies of its impact on protein trafficking. The studies covered in this review have primarily conducted experiments on polarized epithelial cells grown on a permeable support, or in 2D. While 2D studies can provide valuable insights into the trafficking of proteins in polarized epithelial cells, 3D studies where epithelial cells are cultured on extracellular matrix components (e.g., Matrigel), or *in vivo* studies are more physiologically relevant (Singh et al., 2013; Singh et al., 2015).

**FIGURE 2:**
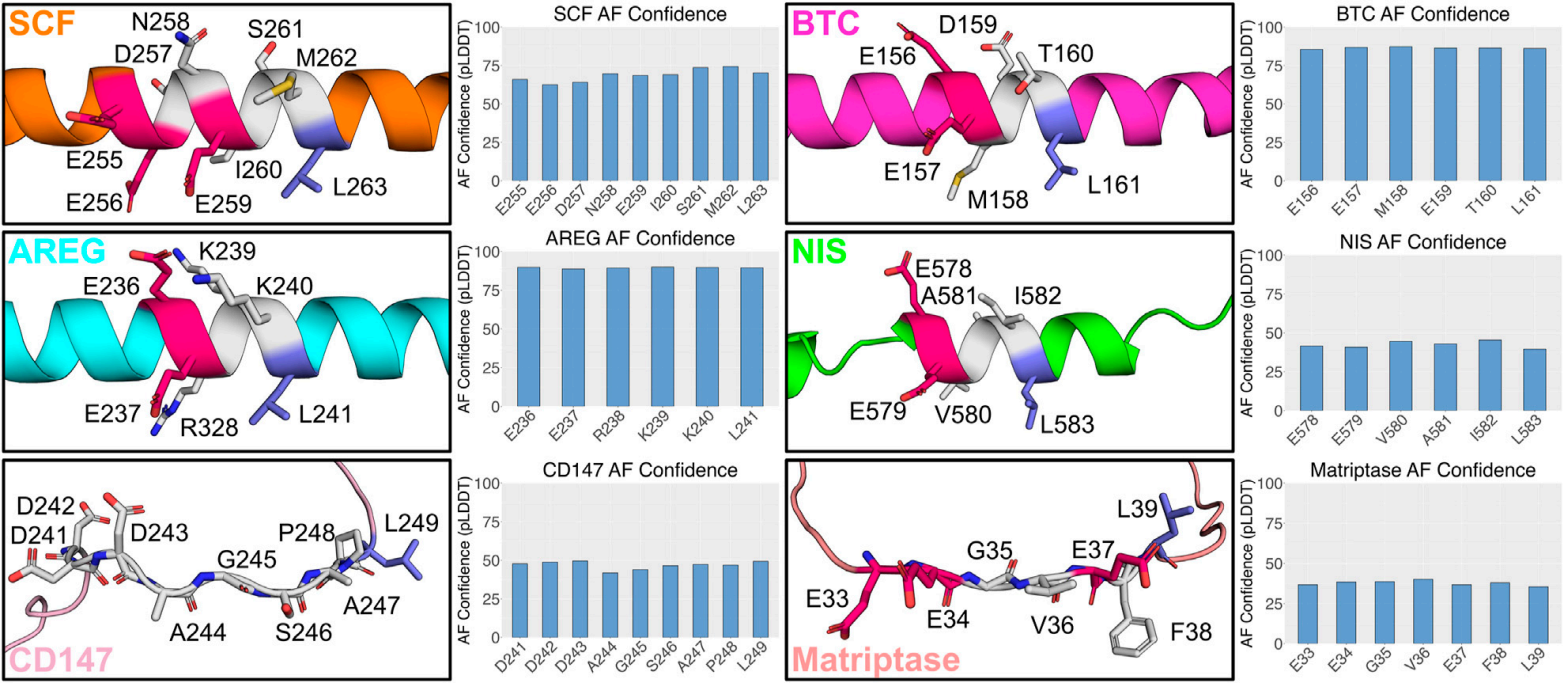
Predicted structure of identified monoleucine-based sorting motifs. AlphaFold version 2.3.2 was run locally with the full data pipeline to generate predicted 3D structures and confidence values for monoleucine-base sorting motif based on the human sequence for each protein (Jumper et al., 2021). The top ranked AlphaFold structure is presented for each protein. Glutamic acid residues are highlighted in the 3D structure in dark pink. Leucine residues are highlighted in dark blue.

We conducted a structural analysis for all the proteins described above containing the monoleucine-based sorting motif using AlphaFold. Among those, AREG, BTC, SCF, and NIS are predicted to have a helical structure in the region containing the monoleucine-based sorting motif, with varying degrees of confidence (Figure 2). AREG, BTC, and NIS all have three intervening residues between the monoleucine and acidic amino acid cluster. While SCF has five intervening residues, it does have a glutamic acid residue four residues N-terminal to the leucine. This matches the spacing between the leucine and glutamic acid in AREG, BTC, and NIS and suggests it may play a role in shaping the motif. CD147, with five intervening amino acids, and Matriptase, with four intervening amino acids, have a predicted disordered structure in the region of their monoleucine-based sorting motif (Figure 2). These analyses highlight structural heterogeneity within the monoleucie-based basolateral sorting motif, and a potential for differential binding to basolateral sorting adaptors. A better understanding of the impact of the intervening amino acids on the structure of the monoleucine-based sorting motif will better inform the function and interactions of these proteins, including the role of AP-1B in their trafficking.
